# Relationship between Uric Acid to High Density Lipoprotein Cholesterol Ratio and Nonalcoholic Fatty Liver Disease in Nonoverweight/Obese Patients with Type 2 Diabetes

**DOI:** 10.1155/2023/2513175

**Published:** 2023-08-01

**Authors:** Yuliang Cui, Zhenzhen Qu, Wenmei Hu, Haiyan Shi

**Affiliations:** Department of Endocrinology, Qilu Hospital of Shandong University Dezhou Hospital, Dezhou 253000, China

## Abstract

**Aims:**

To investigate the relationship between uric acid to high-density lipoprotein cholesterol ratio (UHR) levels and nonalcoholic fatty liver disease (NAFLD) in nonoverweight/obese patients with type 2 diabetes.

**Methods:**

A retrospective study was designed including a total of 343 inpatients with type 2 diabetes whose BMI<24 kg/m^2^. The population was divided into three groups as the UHR tertiles. Logistic regression analysis was performed to estimate odds ratios (ORs) of UHR for NAFLD. ROC curve analysis was used to estimate the diagnostic value of UHR for NAFLD.

**Results:**

The prevalence rat of NAFLD enhanced progressively from the tertile 1 to tertile 3 of UHR (30.70% vs. 56.52% vs. 73.68%). Logistic regression analysis showed that participants in the higher UHR groups, compared with those in the first tertile group, had higher occurrence risks for NAFLD. The positive association between UHR and NAFLD was independent of age, BMI, blood pressure, hepatic enzymes, and other components of metabolic disorders. ROC curve analysis showed that the area under curve (AUC), sensitivity, and specificity for UHR were 0.697, 0.761, and 0.553, respectively.

**Conclusions:**

In type 2 diabetic patients without overweight or obesity, UHR is significantly associated with NAFLD and can be used as a novel and useful predictor for NAFLD onset.

## 1. Introduction

Nonalcoholic fatty liver disease (NAFLD) has become one of the most frequent liver diseases with increased morbidity which may be up to 25% in general population globally [1]. The pathological mechanism of NAFLD is characterized by abnormal lipid deposition in hepatic cells, excluding excessive drinking, drugs, and other chronic liver disease such as viral hepatitis [2]. NAFLD can cause a large spectrum of hepatic lesions starting with simple liver steatosis [3]. Then, a complex pathological process is triggered involving chronic hepatitis, hepatic fibrosis, and liver cellular injury, eventually developing to liver cirrhosis, even to liver failure and hepatic carcinoma [4]. In addition, the health damages of NAFLD also spread to extra-hepatic organs. NAFLD has been confirmed to be positively associated with many chronic diseases such as atherosclerotic cardiovascular disease (ASCVD) [5], chronic kidney disease [5], and cancers [6]. The association between NAFLD and type 2 diabetes (T2DM) has also been well revealed [7]. Subjects with NAFLD are more likely to suffer from type 2 diabetes than those without NAFLD [8]; meanwhile, NAFLD is fairly common in type 2 diabetic individuals, affecting 28–70% of the population [9]. More importantly, coexistent NAFLD significantly increases the morbidity and mortality of diabetic complications including cardiovascular events [10], diabetes nephropathy, and diabetes retinopathy [11]. In addition, liver fat accumulation has been indicated to be relevant to reduced insulin sensitivity which could aggravate the metabolism disorders of glucose and lipids [12]. The huge influences of NAFLD on health require an early identification of NAFLD in type 2 diabetic patients.

Most of the patients with T2DM are accompanied by overweight or obesity which is widely known as the major risk element for the onset and development of NAFLD [13]. In type 2 diabetics, the presence of obesity was highly suggested to sift for NAFLD according to EASL guideline [14]. However, our previous study showed that NAFLD was also common in nonobese type 2 diabetic patients, and the degree of metabolic disorders and insulin resistance was more serious in patients with both T2DM and NAFLD [15]. Nonetheless, the study on the NAFLD, in type 2 diabetic patients without overweight or obesity, is limited. The screening predictors for NAFLD in those patients have not been well clarified. In some previous research studies, serum uric acid was found to be positively associated with the NAFLD risk independent of components of metabolic syndrome in diabetic populations [16, 17]. HDL cholesterol is an important part of plasma lipid profile. Decreased serum HDL-cholesterol levels have been indicated to be associated with a worse metabolic status [18]. A combination of these two metabolic factors is serum uric acid to HDL-cholesterol ratio (UHR) which has recently attracted increasing attentions as a valuable biomarker for metabolic disorders [19], incident ischemic heart disease [20], and diabetic control [21]. A small amount of studies have found a strong relationship between UHR and nonalcoholic fatty liver disease (NAFLD) [22, 23]; however, no study has concerned the association between UHR and NAFLD in the type 2 diabetic population, especially in nonoverweight/obese diabetic subjects. Thus, we aimed to examine whether UHR was independently correlative with the prevalence of NAFLD in type 2 diabetic patients without overweight or obesity.

## 2. Materials and Methods

### 2.1. Research Population

The objects collected in our research were patients hospitalized in the Endocrine Department, Qilu Hospital of Shandong University Dezhou Hospital, from 2016 to 2022. The participants were selected with these criteria: (1) individuals with type 2 diabetes whose BMI was not more than 24 kg/m^2^ according to the guidelines for overweight/obesity in China [24] in which the criteria of overweight and obesity were BMI ≥ 24 kg/m^2^ and BMI ≥ 28 kg/m^2^, respectively, for Chinese adults, (2) those without large quantities of alcohol intake (alcohol intake<30 g/d for males and<20 g/d for females), and (3) those without a history of other hepatic diseases including viral hepatitis and autoimmune hepatitis. The exclusive criteria were as follows: (1) those with seriously acute or chronic complications such as diabetes ketoacidosis, infection, myocardial infarction, diabetic gangrene, cardiac insufficiency, and kidney failure, (2) those with stringent state, gestation, trauma, and other situations affecting the results, and (3) those using any medicine that can affect the levels of uric acid and HDL cholesterol such as benzbromarone, febrista, statins, and insulin. Ultimately, 343 inpatients were enrolled in the investigation.

We preformed this research in accordance with the regulations of the Helsinki Declaration. Ethical approval was obtained for this cross-sectional study from the Ethics Committee of Qilu Hospital of Shandong University Dezhou Hospital. Written informed consents were provided by all participants.

### 2.2. Physical Measurements and Biochemical Examinations

The anthropometric indicators such as height, body weight, systolic blood pressure (SBP), and diastolic blood pressure (DBP) were measured following the standardized approaches. The body mass index (BMI) was calculated as weight (kg)/height (m^2^). The general information of the population including age, gender, and medical history was recorded carefully. Fasted blood samples were collected after overnight (for more than 8 hours) and were detected in the department of clinical laboratory, using the automatic biochemical analyzer. The biochemical parameters including serum uric acid (SUA), alanine aminotransferase(ALT), aspartate transferase(AST), *γ*-glutamyl transpeptidase (*γ*-GGT), high-density lipoprotein cholesterol (HDL), fasting blood glucose (FBG), total cholesterol (TC), triglycerides (TG), low-density lipoprotein cholesterol (LDL), blood urea nitrogen (BUN), and serum creatinine (Cr) were measured and recorded by trained technicians. Serum fasting insulin (FIN) was measured with chemiluminescence assay. UHR was obtained dividing serum uric acid levels by HDL-cholesterol levels. Homeostasis model assessment of insulin resistance (HOMA-IR) was used to assess the insulin resistance, which was calculated as fasting insulin (uIU/mL) × fasting glucose (mmol/L)/22.5.

### 2.3. Examination of Fatty Liver

Liver biopsy was not selected due to its invasive characteristic, and the imaging tool was used to determine the presence of fatty liver based on previous study [22]. An ultrasound scan of the liver was performed for each participant in the department of ultrasound. The diagnosis of NAFLD was based on typical image changes according to the criteria established by the Chinese Association of Liver Disease [25]: (1) the ultrasound beam enhancement in liver antefield, (2) ultrasound beam attenuation in liver far field, and (3) poor visualization of intrahepatic structures, after excluding other forms of hepatic diseases.

### 2.4. Statistical Analyses

Quantitative variables corresponding to normal distribution were displayed by means ± standard deviations, and those with skewed distribution were presented by medians (intertertile ranges). Qualitative data were expressed as numbers and frequencies. The population was divided into NAFLD group and non-NAFLD group. Independent sample *T* test (for normal distribution) and Wilcoxon rank sum test (for skewed distribution) were used to assess differences between these two groups. To evaluate the relationship between UHR and NAFLD, all individuals were categorized into three groups according to the tertiles of UHR: UHR tertile 1, <211.21 *μ*mol/mmol; UHR tertile 2, 211.21–295.04 *μ*mol/mmol; and UHR tertile 3, >295.04 *μ*mol/mmol. The comparisons of NAFLD incidences among the tertiles were evaluated by the chi-square test. Logistic regression analysis was conducted to investigate the association of UHR tertiles with the risk of NAFLD by building three test models (model 1: without adjustment; model 2: adjusting for age, gender, BMI, SBP, and DBP; and model 3: adjusting for age, gender, BMI, SBP, DBP, FIN, ALT, AST, *γ*-GGT, TC, TG, LDL, FBG, BUN, Cr, and HOMA-IR), and the unadjusted and adjusted odds ratios (ORs) as well as 95% confidence intervals were given. Furthermore, the receiver operator characteristic (ROC) curve analysis was applied to estimate the function of UHR for the detection of NAFLD, and the susceptibility and specificity as well as cut-off point were calculated. All analyses were conducted using SPSS 21.0. The *P* values (two-sided) less than 0.05 were supposed to be statistically significant.

## 3. Results

### 3.1. General Characteristics of Research Subjects

There were 209 males and 134 females among these 343 type 2 diabetic participants with average age of 50.96 years and median BMI of 22.68 kg/m^2^. The total prevalence of NAFLD was 53.6% (184 persons). Physical and biochemical indexes on the basis of the presence of NAFLD are presented in Table 1. Patients with NAFLD were more likely to be heavier and had significantly enhanced levels of SBP, DBP, FIN, ALT, AST, *γ*-GGT, TC, TG, FBG, LDL-C, SUA, and HOMA-IR and decreased levels of HDL compared to those without NAFLD. Moreover, sensibly higher UHR levels were observed in individuals with NAFLD than in non-NAFLD subjects (294.80 ± 99.90 versus 228.67 ± 88.08, *P* < 0.001) (Table 1).

### 3.2. Correlation between UHR and the NAFLD Risk

The NAFLD prevalence rat in UHR tertile 1 group was 30.70% and increased to 56.52% and 73.68% in the tertile 2 and tertile 3 groups, respectively (Table 2). The unadjusted ORs for NAFLD in the tertile 2 group and tertile 3 group were 2.93 (95% CI: 1.770–5.04) and 6.32 (95% CI: 3.55–11.24) compared with tertile 1 (Table 3). After adjusting for gender, age, BMI, SBP, and DBP (model 2), the ORs for NAFLD in tertile 2 and tertile 3 were 2.58 (95% CI: 1.42–4.71) and 6.32 (95% CI: 3.24–12.32) (Table 3). With further adjustment for gender, age, BMI, SBP, DBP, FIN, ALT, AST, *γ*-GGT, TC, TG, LDL, FBG, BUN, Cr, and HOMA-IR (model 3), the ORs of NAFLD remained significantly increased for tertile 2 (OR = 2.17, 95% CI: 1.06–4.48) and tertile 3 (OR = 3.73, 95% CI: 1.53–9.09) (Table 3).

### 3.3. The Detective Ability of UHR for NAFLD

**Figure 1:**
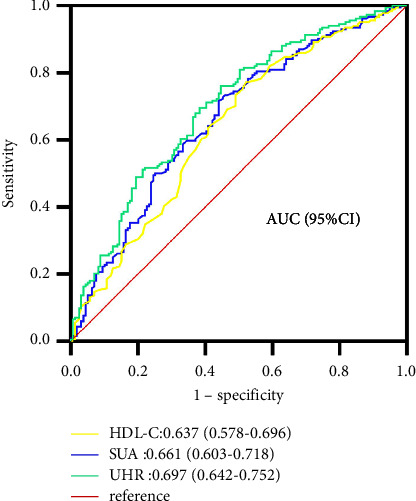
ROC curves for UHR, compared to SUA and HDL-C alone. The indicative ability of UHR is greater compared to that of SUA or HDL-C alone according to its AUC.

ROC curve analysis was used to assess the detective ability of UHR for NAFLD occurrence. The area under curve (AUC) of UHR was 0.697 which was higher than that of SUA (0.661) and HDL (0.637) (Figure 1). The sensitivity and specificity of UHR were 0.761 and 0.553, respectively (Table 4). The Youden index and cut-off point of UHR were 0.314 and 222.26, respectively (Table 4).

## 4. Discussion and Conclusions

Our research confirmed that increased UHR levels were independently relevant to higher risks of NAFLD in nonoverweight/obese type 2 diabetics. UHR can be considered as a useful biomarker for NAFLD in nonoverweight/obese patients with type 2 diabetes.

Nonalcoholic fatty liver disease and type 2 diabetes are both the most common chronic conditions worldwide driving huge economic and health burdens [26, 27]. The relationship between NAFLD and type 2 diabetes has been widely clarified. The occurrence of NAFLD is quite universal in population with T2DM, with the maximal morbidity at 70% for NAFLD in Europe and the minimum morbidity at 30% in Africa [28]. Moreover, the coexistence of T2DM and NAFLD significantly increases the risks of diabetic complications. Targher et al. [10] showed that NAFLD independently enhanced the risk of the development of cardiovascular diseases by 1.87-fold in type 2 diabetic individuals. In other studies, hypertension and metabolic syndrome were indicated to be more prevalent in subjects with both T2DM and NAFLD than those having T2DM only [29]. Nevertheless, most of previous studies were conducted in patients with T2DM without excluding the impact of obesity. In fact, the beginning of type 2 diabetes appears mostly in obese population, and the incidence of overweight and obesity in people with T2DM is up to 50.9%–98.6% [30]. Overweight and obesity are well known to be interlinked risk factors for both NAFLD prevalence and development [31]. Therefore, it is easy to identify NAFLD in overweight and obese patients. However, the exploration for NAFLD among type 2 diabetics without overweight or obesity is very scarce. Our study showed that, among population with T2DM whose BMI < 24 kg/m^2^, NAFLD covered approximately half of the participants and was associated with more serious metabolic disorders and insulin resistance, highlighting the importance of early identification of NAFLD in these patients.

Our research confirmed that UHR had significant ability for detecting NAFLD in nonoverweight/obese type 2 diabetics. We observed that the UHR levels were significantly increased in patients with NAFLD compared to those without NAFLD. In addition, the prevalence of NAFLD increased progressively from the lowest tertile to the highest tertile of UHR. Logistic regression analysis showed that participants in the higher UHR tertiles, compared with those in the first tertile, had higher risks for NAFLD, suggesting that individuals with increased UHR are more possible to have NAFLD compared with those with reduced UHR. This association between UHR and NAFLD risks was independent of multiple confounding factors including age, gender, BMI, blood pressure, hepatic enzymes, and other components of metabolic syndrome. Furthermore, the ROC analysis showed that UHR had a significant predictive power for the onset of NAFLD which was better than SUA and HDL alone. These findings suggest that UHR can serve as a potential biomarker for NAFLD in nonoverweight/obese type 2 diabetic populations.

Uric acid is the end product of purine metabolism. Various chronic diseases, such as cardiovascular disease (CVD) [32], insulin resistance, type 2 diabetes, and high blood pressure [33], are frequently found to be related to uric acid increase. Previous studies have confirmed that SUA was positively associated with NAFLD after adjusting for multiple factors [34]. Hyperuricemia can induce the development of insulin resistance, mitochondrial oxidative stress, and inflammation response, which are all risk factors for liver fibrosis [35, 36]. HDL cholesterol plays important roles in metabolic syndrome and is also closely associated with NAFLD [37]. One of the lipid profiles in subjects with NAFLD is characterized by reduced HDL-cholesterol [38]. Low HDL cholesterol was found to be associated with a worse metabolic status, which significantly increased the risk of NAFLD [39]. The UHR, which is calculated dividing serum uric acid levels by HDL-cholesterol, has recently attracted increasing attentions. UHR has been showed to be increased in metabolic syndrome and suggested as a more sensitive predictor of metabolic syndrome than every other markers of this syndrome [40]. Aktas et al. [21] suggested that UHR could serve as a useful predictor of diabetic control in type 2 diabetic males, owning to its positive association with HbA1c and FPG levels. A small number of previous studies have shown that UHR was positively correlated with NAFLD [22]. Zhang et al. [23] suggested that UHR was independently related to an enhanced risk of NAFLD, in which when the levels of UHR increased by 1%, the risk for NAFLD occurrence increased by 10.5%. Our research presented for the first time that, in nonoverweight/obese type 2 diabetic subjects, higher UHR was strongly and independently associated with an increased risk of NAFLD. UHR can be measured as a useful and economical predictor for the onset of NAFLD.

There are several shortcomings in this study. First, this was a single-center study and the sample size was relative small. Thus, the conclusion may not be universal. Second, patients with NAFLD in the present study were diagnosed by ultrasound, which is relatively insensitive for the degree of liver fibrosis. Hence, association between UHR and the severity of hepatic steatosis could not be determined. Third, there was no consideration for other obesity-related factors including waist circumstance and body fat content. Therefore, some obese patients with normal BMI were not been identified. Therefore, well-designed cohort studies with a larger sample should be conducted to further explore the screening capacity of UHR for NAFLD in nonoverweight/obese patients with T2DM.

## 5. Conclusion

Our study demonstrated that, in nonoverweight/obese patients with type 2 diabetes, UHR levels were positively associated with NAFLD occurrence, independent of hepatic enzymes and multiple metabolic risk factors. UHR is a reliable predictor to stratify the higher risks of NAFLD in type 2 diabetes patients without overweight or obesity.

## Figures and Tables

**Table 1:** Clinical and biochemical characteristics of study subjects and the differences of factors between patients with non-NAFLD and NAFLD.

General indexes	All patients (*n* = 343)	NAFLD (*n* = 184)	Non-NAFLD (*n* = 159)	*T*/*Z*	*P*
Age (yr)	50.96 ± 11.67	50.85 ± 11.70	51.08 ± 11.66	0.18	>0.05^*∗*^
BMI (kg/m^2^)	22.68 (21.80–23.30)	22.94 (22.20–23.45)	22.21 (20.70–22.96)	−5.72	<0.001^*∗*^
SBP (mmHg)	131.94 ± 17.68	135.19 ± 17.88	128.18 ± 16.74	−3.71	<0.001^*∗*^
DBP (mmHg)	81.89 ± 11.55	84.15 ± 11.14	79.27 ± 11.49	−3.97	<0.001^*∗*^
FINS (pmol/l)	29.54 (16.76–47.05)	33.93 (20.24–54.50)	24.02 (13.23–39.97)	−3.99	<0.001^*∗*^
ALT (IU/L)	19.4 (15–30.45)	24.5 (16.8–36.8)	16.9 (13–21.83)	−5.88	<0.001^*∗*^
AST (IU/L)	20.07 (16.3–25.7)	22 (17.4–29.1)	18.4 (15.08–23.33)	−4.15	<0.001^*∗*^
*γ*-GGT (IU/L)	29.25 (19.48–44.08)	33.6 (23.08–50.63)	22 (16.28–38.08)	−5.13	<0.001^*∗*^
TC (mmol/L)	5.14 ± 1.77	5.35 ± 2.10	4.89 ± 1.25	−2.42	<0.05^*∗*^
TG (mmol/L)	1.30 (0.87–1.93)	1.68 (1.22–2.46)	0.94 (0.72–1.35)	−8.85	<0.001^*∗*^
HDL (mmol/L)	1.15 ± 0.27	1.09 ± 0.23	1.22 ± 0.29	4.57	<0.001^*∗*^
LDL (mmol/L)	3.21 ± 0.89	3.32 ± 0.84	3.08 ± 0.92	−2.54	<0.05^*∗*^
FBG (mmol/L)	7.92 (6.26–10.28)	8.53 (6.59–10.80)	7.30 (6.01–9.93)	−2.72	<0.01^*∗*^
BUN (mmol/L)	5.30 ± 1.47	5.21 ± 1.41	5.41 ± 1.54	1.22	>0.05^*∗*^
Cr (*μ*mol/L)	63.88 ± 18.23	65.42 ± 16.01	62.11 ± 20.41	−1.67	>0.05^*∗*^
SUA (*μ*mol/L)	287.58 ± 82.82	307.48 ± 80.22	264.56 ± 80.00	−4.94	<0.001^*∗*^
HOMA-IR	10.44 (5.53–18.32)	12.99 (6.96–20.97)	7.45 (4.30–13.62)	−4.69	<0.001^*∗*^
UHR (*μ*mol/mmol)	264.14 ± 100.07	294.80 ± 99.90	228.67 ± 88.08	−6.45	<0.001^*∗*^

SBP: systolic pressure; DBP: diastolic pressure; FINS: fasting insulin ALT: alanine aminotransferase; AST: aspartate transferase; *γ*-GGT: *γ*-glutamyl transpeptidase; TC: total cholesterol; TG: triglycerides; HDL: high-density lipoprotein; LDL: low-density lipoprotein; FBG: fasting blood glucose; BUN: blood urea nitrogen; Cr: creatinine; SUA: serum uric acid; UHR: uric acid to HDL cholesterol ratio; HOMA-IR: insulin resistance index. The *t* test (for normal distribution) and Wilcoxon rank sum test (for skewed distribution) with different samples were adopted for comparison between groups. ^*∗*^*P* < 0.05 was considered as statistically significant difference.

**Table 2:** Differences of the prevalence rat of NAFLD among UHR tertiles.

UHR quartile	Total	NAFLD	Prevalence rat (%)	*X* ^2^	*P* value
Quartile 1	114	35	30.70		
Quartile 2	115	65	56.52		
Quartile 3	114	84	73.68	42.924	<0.001^*∗*^

UHR: uric acid to HDL cholesterol ratio. UHR tertile 1, <211.21 *μ*mol/mmol; UHR tertile 2, 211.21–295.04 *μ*mol/mmol; UHR tertile 3, >295.04 *μ*mol/mmol. The chi-square test was used to evaluate the comparisons in NAFLD prevalence among the tertiles. ^*∗*^*P* < 0.05 was considered as statistically significant difference.

**Table 3:** Summary of regression analysis of the correlation between UHR quartiles and NAFLD.

	Quartile 1 (*n* = 114)	Quartile 2 (*n* = 115)	Quartile 3 (*n* = 114)
Model 1 *P*	1	2.93 (1.770–5.04) <0.001^*∗*^	6.32 (3.55–11.24) <0.001^*∗*^
Model 2 *P*	1	2.58 (1.42–4.71) <0.01^*∗*^	6.32 (3.24–12.32) <0.001^*∗*^
Model 3 *P*	1	2.17 (1.06–4.48) <0.05^*∗*^	3.73 (1.53–9.09) <0.01^*∗*^

UHR: uric acid to HDL cholesterol ratio. UHR tertile 1, <211.21 *μ*mol/mmol; UHR tertile 2, 211.21–295.04 *μ*mol/mmol; UHR tertile 3, >295.04 *μ*mol/mmol. Model 1: unadjusted analyses. Model 2: adjusted for gender, age, BMI, SBP, and DBP; Model 3: adjusted for gender, age, BMI, SBP, DBP, FIN, ALT, AST, *γ*-GGT, TC, TG, LDL, FBG, BUN, Cr, and HOMA-IR.

**Table 4:** ROC curve analysis for UHR, SUA, and HDL-C.

Factor	AUC	95% CI	Sensitivity	Specificity	Youden index	Cut-off point	*P* value
UHR	0.697	0.642–0.752	0.761	0.553	0.314	222.26	<0.001^*∗*^
SUA	0.661	0.603–0.718	0.717	0.559	0.277	259.35	<0.001^*∗*^
HDL-C	0.637	0.578–0.696	0.478	0.772	0.249	1.22	<0.001^*∗*^

UHR: uric acid to HDL cholesterol ratio; SUA: serum uric acid; HDL: high-density lipoprotein; AUC: area under curve.

## Data Availability

The data are unavailable because the data sharing should be agreed by the authors' ethics committee. In addition, the authors will conduct further studies based on these data.
